# Sociogenesis in Unbounded Space: Modelling Self-Organised Cohesive Collective Motion

**DOI:** 10.1088/1478-3975/acc4ff

**Published:** 2023-03-28

**Authors:** Zohar Neu, Luca Giuggioli

**Affiliations:** Department of Engineering Mathematics, University of Bristol, Bristol, BS8 1TW, United Kingdom; Bristol Centre for Complexity Sciences, University of Bristol, Bristol, BS8 1UB, United Kingdom

**Keywords:** sociogenesis, dominance hierarchy, collective motion, self-organisation, interacting random walk

## Abstract

Maintaining cohesion between randomly moving agents in unbounded space is an essential functionality for many real-world applications requiring distributed multi-agent systems. We develop a bio-inspired collective movement model in 1D unbounded space to ensure such functionality. Using an internal agent belief to estimate the mesoscopic state of the system, agent motion is coupled to a dynamically self-generated social ranking variable. This coupling between social information and individual movement is exploited to induce spatial self-sorting and produces an adaptive, group-relative coordinate system that stabilises random motion in unbounded space. We investigate the state-space of the model in terms of its key control parameters and find two separate regimes for the system to attain dynamical cohesive states, including a Partial Sensing regime in which the system self-selects nearest-neighbour distances so as to ensure a near-constant mean number of sensed neighbours. Overall, our approach constitutes a novel theoretical development in models of collective movement, as it considers agents who make decisions based on internal representations of their social environment that explicitly take into account spatial variation in a dynamic internal variable.

## 1 Introduction

Systems exhibiting self-organised collective motion and spatial sorting are widespread across scales in nature [25], from flocking [22, 6] and separation with respect to social or physical characteristics [14, 21, 1] to territorial segregation [19, 15]. Of the existing models of collective movement, little attention has been given to the role of *sociogenesis* [26], the theory of how socio-spatial structures form as a result of agent interactions. In nature, however, there is a strong interplay between social dynamics, movement and space-use behaviour. Annular socio-spatial patterns have been widely observed in primate dominance hierarchies, where high ranking individuals occupy central locations in the group, while low ranking ones are found at the peripheries [18, 14].

While existing spatially explicit models of dominance hierarchy formation [9, 12, 8] generate correlations between social ranks and spatial centrality, they are restricted to agents that are *reactive* to social information at the *microscopic* scale. The work in [9] analysed the density-driven phase transition for the emergence of condensed clusters of agents, but did not study the mechanisms with which microscopic scale dynamics lead to cluster formation. Work in [12, 8] investigated how heterogeneity in agent repulsion generated by micro-scale collisions leads to spatial sorting. A common characteristic of these models is the imposition of periodic boundary conditions to maintain group cohesion. This is not surprising given that, in unbounded domains, cohesive collective random motion is known to require direct [24, 6, 16, 29] or effective [17] attractive interactions that share information at long range.

Here, we present a sociogenesis framework for self-organised cohesive collective motion in unbounded space. We posit that loss of cohesion occurs due to instabilities arising from agents who base their movement decisions on noisy *motion-dependent* information at the microscopic scale. As such, we model agents whose behaviour is determined by their belief state - a coarse-grained representation of the local environment. The belief enables agents to respond to socio-spatial information at the *mesoscopic scale*, which fluctuates at a slower time-scale than the microscopic states it encodes. Importantly, agent beliefs in our model represent spatial variations in internal variables that are not related to motion.

In view of our modelling approach, it is noted that while existing models of flocking [24, 22, 6] also leverage mesoscopic-level social interactions to achieve global collective patterns, internal representations have only made use of information corresponding to agent states that are position and motion-related. As a result, the functional form of the agent beliefs used in these models has been restricted to taking an average of positions and velocities of neighbouring agents. Models of spatial coordination in multi-agent systems (MAS) and reinforcement learning (RL) have similarly focused on using internal representations of spatial information, computing Voronoi area partitions [5] and distributed path selection [4] based on neighbour positions that are directly sensed or propagated through message passing. Other approaches have focused on coordinating agent motion by optimising coarse-grained, position-related metrics over the agent communication network, such as the number of network connections with neighbours [28], or using shared neighbour information [27].

The model presented here offers a generalisation of the mesoscopic coarse-graining procedure by extending the functional form of agent beliefs beyond taking an average. This development is necessary for enabling agents to coordinate using dynamic internal variables that are not motion-related. Since coarse-graining via a simple average leads to a loss of spatial information, coordination of agents using a dynamic internal variable requires a belief which takes into account spatial variation. In this model, a spatially explicit regression model is used for coarse-graining. The form of this generalisation allows for further extension beyond regression to other machine learning approaches.

In fact, this generalisability is essential to the functionality of our model, since different functional forms of the belief generate different global socio-spatial configurations. Here, the beliefs are constrained to induce self-organisation into a desired global concave annular state. We consider a system in 1D space, and use the term *radial* to refer to the line segment extending from the central point to the outer bounds of a given 1D interval, meaning that the desired global state is radially sorted in the social variable.

## 2 Model

In this section, we describe the model in terms of its key variables. Tab. 1 summarises the various symbols in the model with their definitions and parameter values. The system is made up of *N* randomly moving agents on a discrete, unbounded 1D lattice. Time, *t*, is discrete, and each agent, *k*, is described by its position, *x_k_* ∈ ℤ, and an internal variable describing its social rank, *S_k_* ≥ 0. Social rank dynamics are defined through pairwise repulsive interactions inspired by the Bonabeau-Theraulaz-Deneubourg model [3]. When two agents *j* and *k* are co-located, they interact with probability *P_jk_*(*S_j_, S_k_*). If an interaction occurs, the winner and loser are selected according to an outcome probability *Q_jk_*(*S_j_, S_k_*), where *j* wins with probability *Q_jk_*(*S_j_, S_k_*), and *k* with probability *Q_kj_*(*S_k_, S_j_*) = 1 −*Q_jk_*(*S_j_, S_k_*). The social ranks of the winner and loser are increased and decreased by *δ*^+^ and *δ*^−^, respectively. At the mesoscopic scale, these social dynamics produce a spatially explicit social field, *S*(*x, t*), describing the expected value of *S_k_* at position *x* and time *t*.

**Figure 1:**
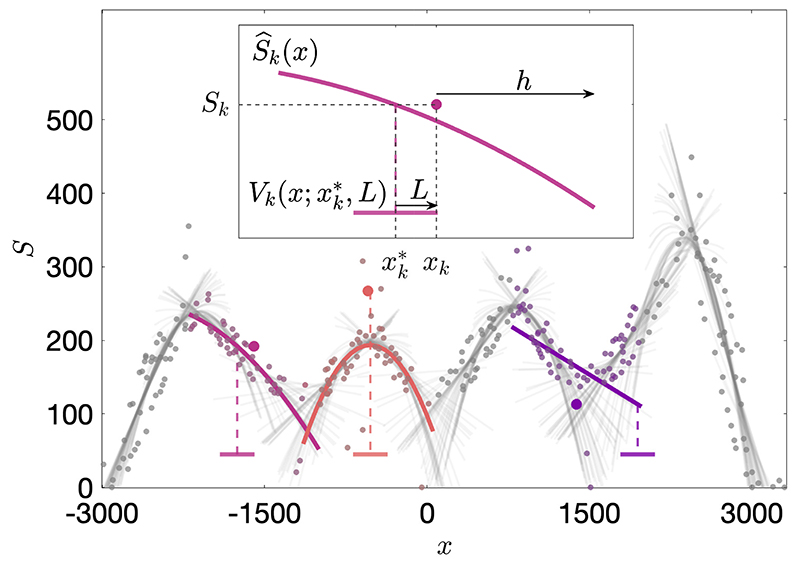
(Colour online) Illustration of the model with *N* = 300 agents after *t* = 1.96 × 10^4^ steps, with initial density *ρ*_0_ = 0.05, social ranks *S_k_*(0) = 0, *k_L_* = 8, *k_h_* = 0.1 and Δ*S_max_* = 100. Main Figure: Each point represents an agent with position, *x_k_*, social rank, *S_k_*, and belief, *Ŝ_k_*(*x*), plotted as a curve of horizontal half-length *h* to illustrate the sensing range. Three selected agents are shown with larger circles, and each of their box potentials, $V_{k}\left(x;x_{k}^{*},L\right)$, is plotted as a bar underneath with center $x_{k}^{*}$ and radius *L*. The left-most and middle selected agents perform second-order polynomial regression. The middle agent defines $x_{k}^{*}$ at the maximum of *Ŝ_k_*(*x*) because its social rank *S_k_* > *Ŝ_k_*(*x*) for all *x*. The right-most agent senses a minimum in *S*(*x, t*), and so performs a first-order polynomial regression. Inset: Detailed view of a single agent.

Our model makes several modifications to the original formulation in [3], to ensure that the generated hierarchies are stable. We define a bounded interaction probability, *P_jk_*(*S_j_, S_k_*) = *H*_0_(Δ*S_max_* − |*S_j_* − *S_k_*|), where *H*_0_(*S*) is the Heaviside step function with *H*_0_(0) = 0, so agents only interact if the difference in their social ranks is below the threshold, |*S_j_* − *S_k_*| < Δ*S_max_*. Without this threshold, higher density regions experience a faster growth of social ranks due to higher encounter rates, and the steepness of *S*(*x, t*) may become unbounded. On the other hand, when Δ*S_max_* is too small, the system does not have enough time to socially differentiate and attain the concave annular state. The hand-tuned value of Δ*S_max_* = 100 is used in all simulations. Furthermore, a deterministic outcome probability is used so that the higher rank agent always wins, *Q_jk_*(*S_j_, S_k_*) = *H*_1/2_(*S_j_* − *S_k_*), where *H*_1/2_(*S*) is the Heaviside step function with *H*_1/2_(0) = 1/2, so that a winner is selected at random when *S_j_* = *S_k_*. We set *δ*^+^ = 1 and *δ*^−^ = 0, and there is no relaxation of social ranks towards zero in our model. Fig. 1 shows a schematic of the model, demonstrating the belief computation and navigation procedures described below.

Sensing and Belief Computation: A communication region with radius *h* is defined around each agent, *k*, within which the agent can sense the positions, *x_j_*, and social ranks, *S_j_*, of neighbours, *j*. At every time-step, each agent performs a sensing measurement over this region with probability *r* = 0.01. This makes agent sensing asynchronous and is computationally efficient. An agent computes its belief, *Ŝ_k_*(*x*), from sensed neighbour data using a weighted polynomial regression with functional form *Ŝ_k_*(*x*) = *p*(*x*) + ξ (supplementary material sect. II), estimating the coefficients of the polynomial *p*(*x*). Agents compute *Ŝ_k_*(*x*) as a second-order polynomial, *Ŝ_k_*(*x*) = *β*_0_ + *β*_1_*x* + *β*_2_*x*^2^ + ξ, which captures turning points in *Ŝ*(*x, t*). To destabilise minima and retain a single maximum, a first-order polynomial belief, *Ŝ_k_*(*x*) = *β*_0_ + *β*_1_*x* + *ξ*, is computed instead of a second-order belief whenever a minimum is measured, i.e. *β*_2_ > 0. Each belief has an associated model fidelity, Φ_*k*_ ∈ (−∞, 1], that measures its accuracy, given by

(1) $${\text{Φ}}_{k}=1-\frac{{\text{−}}_{j=1}^{n}{(S_{j}-{\hat{S}}_{k}\left(x_{j}\right))}^{2}}{{\text{−}}_{j=1}^{n}{\left(S_{j}-\bar{S}\right)}^{2}},$$

where *S_j_* are the social ranks of the *n* sensed neighbours of *k*, and $\bar{S}={\text{−}}_{i}S_{i}/n$ is the average of the sensed social ranks. A value of Φ_*k*_ → 1 indicates a good fit, and Φ_*k*_ → −∞ indicates a worse fit (supplementary material sect. II C).

Agent Navigation: Agents perform random walks on the lattice, subject to an individual potential, $V_{k}\left(x;x_{k}^{*},L\right)$, here taken to be the box potential with radius *L* and discrete-valued center, $x_{k}^{*}\in \mathbb{Z}$,

(2) $$V_{k}\left(x;x_{k}^{*},L\right)=\left\{ \begin{array}{l} 0\;\;\text{if}\;\left|x-x_{k}^{*}\right|<L,\\ \infty \;\;\text{if}\;\left|x-x_{k}^{*}\right|\geq L. \end{array}\right.$$

The bounded potential is chosen so as to limit rare events of complete mixing that occur when agents drift into the sensory field of others beyond their nearest neighbours. This ensures a broader region of cohesion stability in the state-space.

Each agent, *k*, computes their potential center, $x_{k}^{*}$, by solving (supplementary material sect. III)

(3) $$x_{k}^{*}(t)=\underset{x}{\text{argmin}}\left(\left|S_{k}(t)-{\hat{S}}_{k}(x)\right|\right),$$

so that $x_{k}^{*}$ is the location that *Ŝ_k_* (*x*) predicts an agent with social rank *S_k_* should occupy. Each $x_{k}^{*}$ is kept discrete by rounding it to the nearest integer. Finally, if the data sensed by an agent is of insufficient quantity or quality for regression to be performed (supplementary material sect. II D), the belief is re-set to zero, *Ŝ_k_* (*x*) = 0, and $x_{k}^{*}$ is not updated.

## 3 Analysis Methods

The mean nearest-neighbour distance is used as a measure of cohesion and dispersion stationarity. This is defined as [29]

(4) $$\text{Δ}(t)=\langle \frac{1}{N}\sum_{j=1}^{N}\underset{k\neq j}{\text{min}}\left(\left|x_{j}(t)-x_{k}(t)\right|\right)\rangle ,$$

where ⟨·⟩ is an ensemble average over different system realisations. The system is considered to be stationary when Δ(*t*) converges to a constant value. We also study the system cohesion in terms of the mean proportion of sensed neighbours, *n_s_*(*t*) ∈ [0, 1], given by

(5) $$n_{s}(t)=\langle \frac{1}{N(N-1)}\overset{N}{\underset{j=1}{\sum }}\overset{N}{\underset{\begin{array}{l} k=1\\ k\neq j \end{array}}{\sum }}𝟙(|x_{j}(t)-x_{k}(t)|\leq h)\rangle ,$$

were the indicator function, i.e.𝟙(*A*) = 1 when *A* is true and 𝟙(*A*) = 0 otherwise, counts the number of sensed neighbours of each agent.

We introduce a global order parameter called the socio-spatial correlation (SSC) that measures how agent social ranks change radially away from the group center of mass. The socio-spatial correlation, *C*(*t*) ∈ [−1, 1], is given by

(6) $$C(t)=\langle \text{Corr}\left(\left|x_{k}(t)-\bar{x}(t)\right|,S_{k}(t)\right)\rangle$$

where *x_k_*(*t*) and *S_k_*(*t*) are the agent positions and social ranks, and $\bar{x}(t)={\text{−}}_{j}\;x_{j}(t)/N$ is the group center of mass. The Spearman rank correlation is used in Corr(·,·), which captures non-linear correlations, and alternative measures do not appreciably change the SSC values obtained. A value of *C*(*t*) = 0 signifies no correlation, while the extreme values of *C*(*t*) = ±1 are attained when social ranks are perfectly radially increasing or decreasing. Hence, *C*(*t*) = −1 signifies convergence to the concave annular state.

We quantify *S*(*x, t*) macroscopically using an integrated sigmoid that we call the shape function,

(7) $$F(x;\alpha ,m,K)=-\frac{2m}{\alpha }\text{ln}\left(1+e^{-\alpha x}\right)-mx+K.$$

This is a concave function that interpolates between quadratic and absolute value functions, with *α, K, m* > 0. While the scale of Eq. (7) depends mostly on *K* and *m*, its shape can be characterised by the parameter *J* = *Kα/m* − ln(4). For large *J, F* (*x*; *α, m, K*) approaches the absolute value function. When *J* ≈ 0, *F* (*x*; *α, m, K*) becomes a quadratic function (supplementary material sect. IV).

## 4 Results

### 4.1 Control Parameters

We investigate the effect of two control parameters, the sensory and box potential radii, *h* and *L*. The value of *h* determines the spatial non-locality of information exchange. The value of *L* determines the encounter rate between neighbouring agents, but is also a source of noise in belief measurements. High values of *L* enable agents to diffuse past the occupation regions of neighbours, perturbing the system spatial sorting, while values of *L* that are too small prevent agents from updating their social ranks through encounters.

The effect of *h* and *L* is relative to the agent density, which determines the proportion of neighbours that can be sensed for a given *h*, as well as the degree of overlap between neighbouring agent occupation regions defined by *L*. The agent density is an output of the system that changes over time, starting from the initially imposed value, *ρ*_0_. We use the initial density to scale the sensory and box potential radii as *h* = *k_h_N/ρ*_0_ and *L* = *k_L_/ρ*_0_. Hence, *k_h_* determines what proportion of the agents can initially be sensed in each radial direction, and *k_L_* determines the expected initial overlap between the occupation regions of neighbouring agents. When *k_h_* = 1, a central agent initially senses all other agents in the system. A value of *k_L_* = 1 initially provides an expected full overlap between neighbouring agent potentials, whereas for *k_L_* < 0.5 there may be gaps between potentials at *t* = 0.

### 4.2 Transient Dynamics

**Figure 2:**
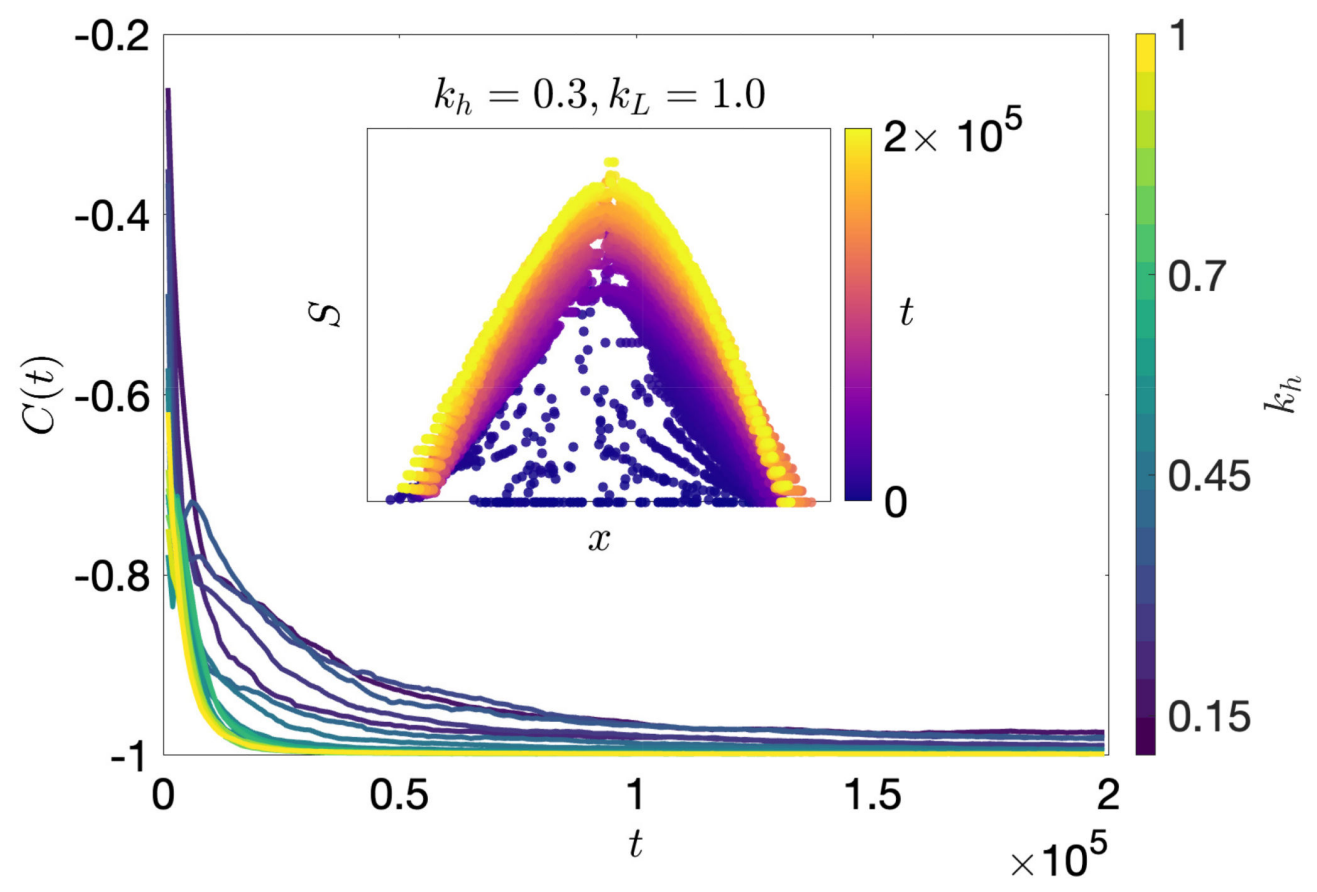
(Colour online) Transient dynamics showing a decrease in the socio-spatial correlation order parameter, *C*(*t*), with time, *t*, signifying convergence to the concave annular state (*C*(*t*) → −1) for different values of the sensory radius scaling, *k_h_*. Increasing *k_h_* for fixed initial density produces faster convergence. Inset: Time evolution of a single realisation for *k_h_* = 0.3 and *k_L_* = 1. Simulations are performed with *N* = 100 agents initialised with *ρ*_0_ = 0.05 and all *S_k_*(0) = 0, Δ*S_max_* = 100, *k_L_* = 1, and *k_h_* ∈ [0.15, 1] increasing in increments of 0.05. Results are averaged over 50 Monte Carlo realisations.

The system dynamics are investigated using Monte Carlo simulations (supplmentary material sect. I). At *t* = 0, agents are initialised with density *ρ*_0_ = *N/A*_0_ by randomly drawing agent positions from a uniform distribution of width *A*_0_, *x_k_*(0) ~ *U* (−*A*_0_/2, *A*_0_/2). Initially, all agents have *S_k_*(0) = 0, $x_{k}^{*}(0)=x_{k}(0)$ and *Ŝ_k_*(*x*) = 0. Fig. 2 shows the system convergence to a concave annular state in terms of the socio-spatial correlation. The inset shows the time evolution of a single simulation, where it can be seen that the system is initially disordered in *x_k_* and *S_k_*, and after some time converges to a concave annular state that continues to grow until all nearest-neighbour agents *j* and *k* have a difference of social ranks equal to |*S_j_* − *S_k_*| = Δ*S_max_*.

The system convergence is explained by the presence of information cascades, which occur in multi-agent settings where actions of agents are influenced by socially acquired information [23]. When all agents are simultaneously adjusting their behaviour in response to that of their neighbours, a high correlation of information is generated between agent states. This information propagates through the agents in the system due to positive feedback effects as follows. Initially, agents measure a weak relationship in the *x_j_* and *S_j_* data of their neighbours, *j*, as represented by the belief, *Ŝ_k_*(*x*). The motion of an agent, *k*, becomes biased according to its belief when it updates its position, *x_k_*, so that *Ŝ_k_* (*x_k_*) ≈ *S_k_*. In doing this, the agent, *k*, implicitly transfers information about its belief to any neighbours who observe its state, (*x_k_, S_k_*). As belief information propagates between agents in the system, neighbours become increasingly more likely to compute the same belief states, ultimately leading to a form of local consensus.

Information cascades result in positive (accurate) or negative (inaccurate) consensus decisions. While convergence occurs as a result of positive information cascades in the system, we also observe negative information cascades that result from inaccurate belief estimates made by agents during early system dynamics [23], as signified by a negative model fidelity, Φ_*k*_ < 0 (supplementary material sect. II C). These early measurement errors may lead to configurations that have radially increasing edges, so that *S*(*x, t*) converges to an ‘N’ or ‘W’ shape.

To address this, we introduce a two-stage dynamics that prevents the use of early, error-prone belief measurements. Our choice here is inspired by biological systems, where multiple time-scales in the formation and maintenance of social ranks have been observed [13]. The two stages are defined by a waiting time, *t_c_*, such that for all *t* < *t_c_*, measurements with an associated model fidelity, Φ_*k*_ < 0, are not retained by agents. This requires agents to have globally synchronised internal clocks, however, the agents’ choice of whether to use their computed belief is local and asynchronous, as this information comes from using the model fidelity, Φ_*k*_, which is locally evaluated.

After *t* > *t_c_*, the use of beliefs with Φ_*k*_ < 0 becomes necessary for system convergence. Agents located at local minima of *S*(*x, t*) tend to compute first-order polynomial beliefs with Φ_*k*_ < 0. In order to destabilise the minima and establish a global maximum characteristic of the concave annular configuration, the agents must make use of these measurements. This use of Φ_*k*_ < 0 measurements when *t > t_c_* does not lead to negative information cascades because, by this time, radially decreasing edges are established which are stable with respect to perturbations caused by erroneous belief measurements (supplementary material sect. V). In subsequent simulations, the hand-tuned value of *t_c_* = 3 × 10^6^ is used, to allow the system sufficient time to develop radially decreasing edges.

### 4.3 Long Time Dependence

**Figure 3:**
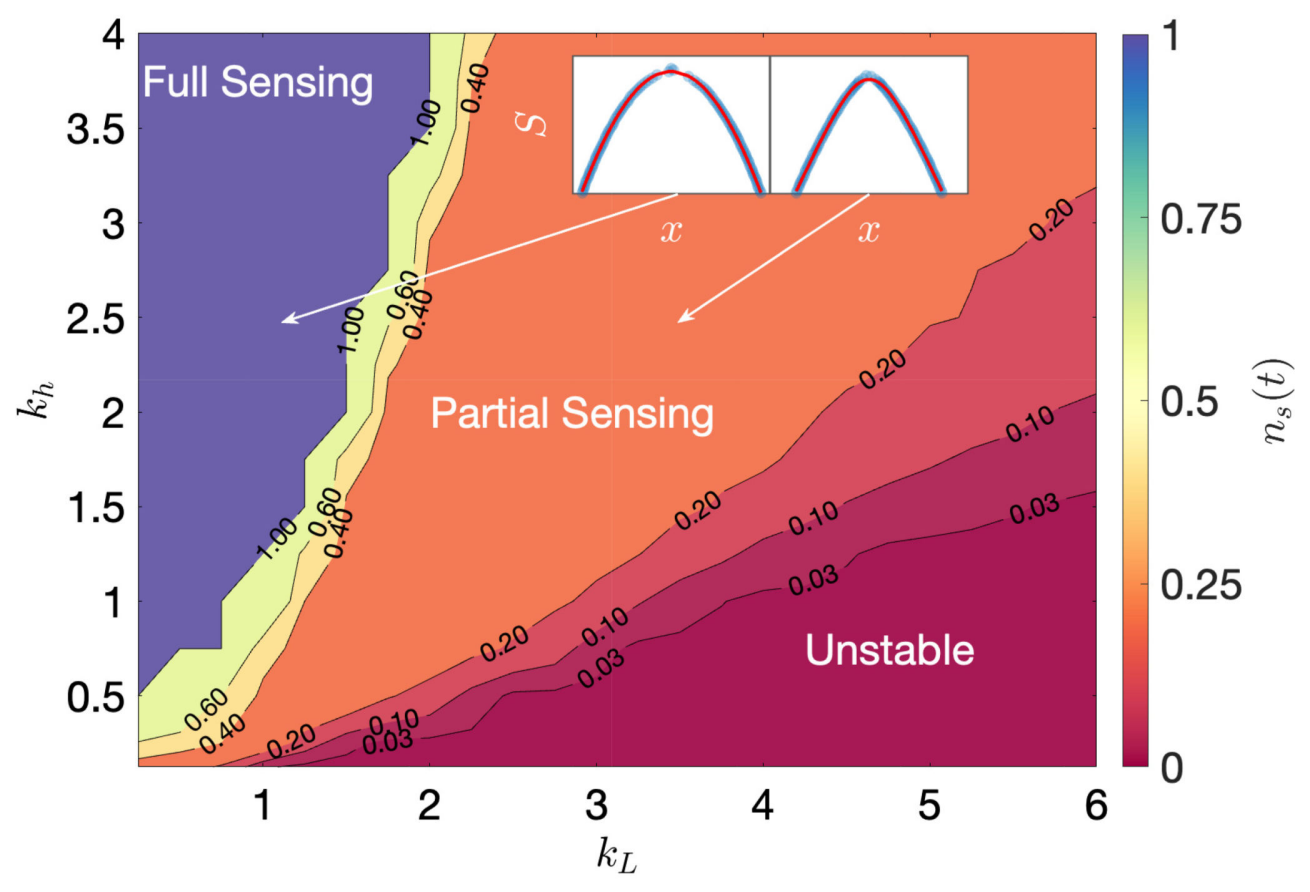
(Colour online) Mean proportion of sensed neighbours, *n_s_*(*t*), at long times (*t* = 10^9^), showing three regimes in the (*k_L_, k_h_*)-state-space. Insets: Average agent configurations (blue points) and the fitted shape function in Eq. (7) (red line) for *k_h_* = 2.5, and with *k_L_* = 1 (*J* = 0.265) on the left and *k_L_* = 3.5 (*J* = 4.88) on the right. Simulation parameters are the same as in Fig. 2, with *t_c_* = 3 × 10^6^. Results are averaged over 10 Monte Carlo realisations.

With Monte Carlo simulations carried out up to *t* = 10^9^, the system exhibits three distinct regimes in the (*k_L_, k_h_*)-state-space, shown in Fig. 3. While the Full and Partial Sensing regimes maintain cohesive distributions of agent positions, the Unstable regime results in a break-up of the agents (supplementary material sect. VI). As can be seen by the contours in Fig. 3, a sharp transition is observed between the Full and Partial Sensing regimes, followed by a more gradual transition between the Partial Sensing and Unstable regimes.

In the Full Sensing regime, the system maintains a similar density to the initial density, *ρ*_0_, with every agent able to sense all others. In the Partial Sensing regime, the system expands by several orders of magnitude beyond its initial density, before reaching a dynamically stable cohesive state. This expansion means that even when agents can initially sense all others in the system, at long times the mean proportion of sensed neighbours in Eq. (5) lies in the range *n_s_*(*t*) ∈ [0.2, 0.4]. In the Unstable regime, the system instead exhibits an expansion that does not stop until the group has completely dispersed and the agent density falls below that required for measurements to take place (supplementary material sect. II D).

**Figure 4:**
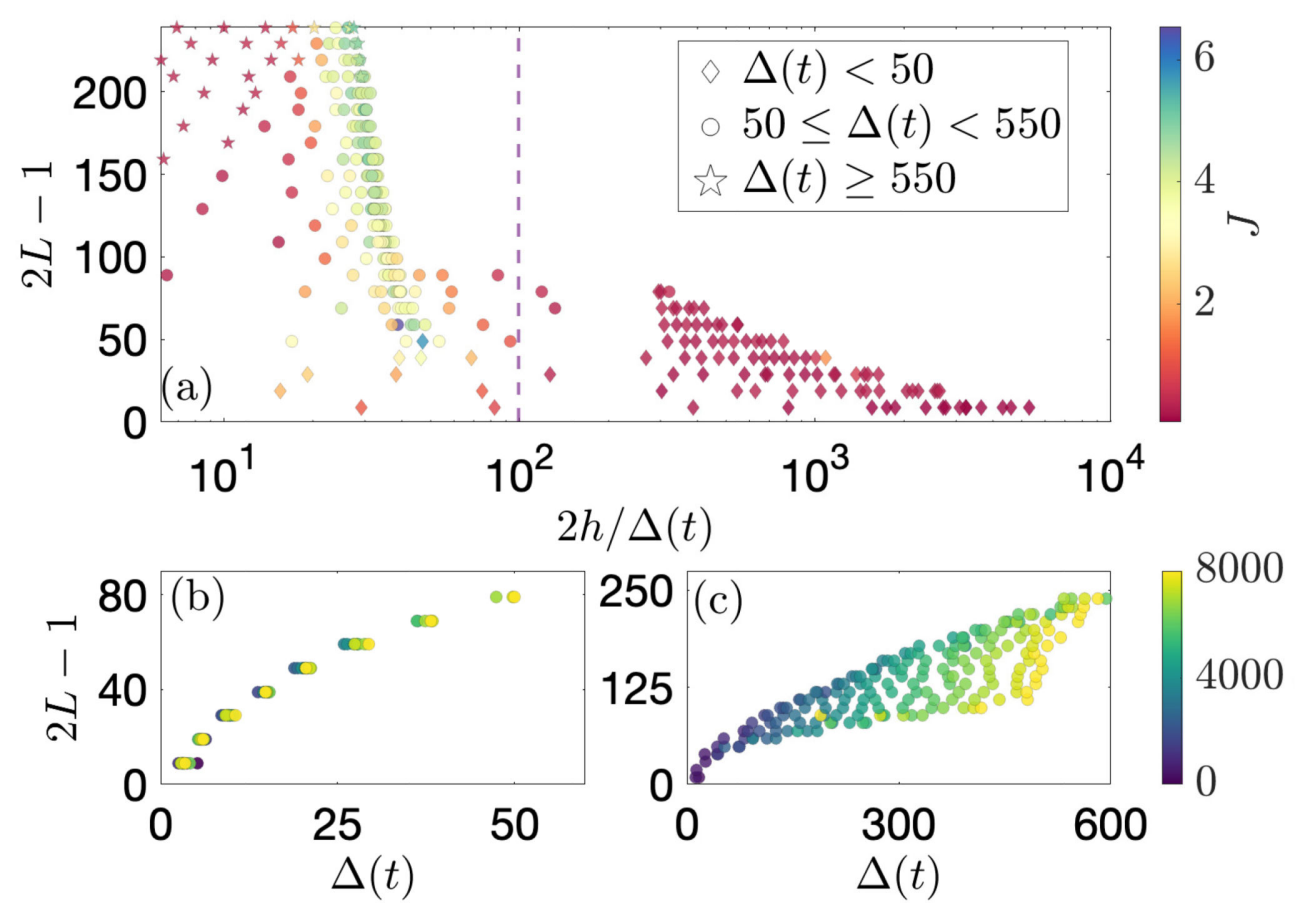
(Colour online) **(a)** Estimated mean number of sensed neighbours, 2*h*/Δ(*t*), for varying box potential lengths, 2*L* − 1. Data points are coloured by the shape parameter, *J*, and the marker shape is determined by the mean nearest-neighbour distance, Δ(*t*). In **(b)** and **(c)**, Δ(*t*) is plotted for varying box lengths, and coloured by the sensor radius, *h*. **(b)** Data from the Full Sensing regime, *n_s_* = 1. **(c)** Data from the Partial Sensing regime, *n_s_* ∈ (0.2, 1). Simulations correspond to those in Fig. 3. Data points with 2*h*/Δ(*t*) < 6 correspond to the Unstable regime and are not plotted.

In Fig. 4(a), we plot the length of the agent occupation region defined by each box potential, 2*L* − 1 (the central site at $x_{k}^{*}$ is only counted once), against the estimated mean number of sensed neighbours, 2*h*/Δ(*t*). The macroscopic shape parameter, *J*, and the microscopic mean nearest-neighbour distance, Δ(*t*), are properties of the system that are not externally imposed, but emerge as a result of the system dynamics following the choice of the microscopic parameters, *h* and *L*. The existence of voids in Fig. 4(a) indicates that only certain values of 2*h*/Δ(*t*) can emerge for each selected box potential length and sensory radius. Namely, there is a jump in the mean number of sensed neighbours between the two cohesive regimes, with the data points around 2*h*/Δ(*t*) = *N* − 1 ≈ 10^2^ belonging to the transition cusp. Similarly, there are no points in the top right-hand region of panel (a), since no states were found in the Full Sensing regime beyond *L* > 45 for all tested values of *h*.

The cluster of points to the right of 2*h*/Δ(*t*) = 10^2^ corresponds to the Full Sensing regime. The emerging mean nearest-neighbour distances, Δ(*t*), lead to fully shared sensory information, so that all agents compute the same belief. Macroscopically, the system assumes a quadratic shape in *S*(*x, t*), as measured by low values of the fitted shape parameter, *J* ≈ 0. The values of Δ(*t*) are independent of *h* in this regime, as shown in panel (b), where the clustering of Δ(*t*) only depends on 2*L* − 1.

In the Partial Sensing regime, the system self-selects values of Δ(*t*) that keep the mean number of sensed neighbours invariant under *L* and *h*. This is shown in panel (a), where data in the Partial Sensing regime is aligned vertically. Macroscopically, the configurations produced here are closer to absolute value functions, with sharper turning points at their center, as measured by higher *J* values. The emerging Δ(*t*) in this regime depend both on *L* and *h*, as seen in panel (c).

## 5 Conclusion

The incorporation of sociogenesis into models of collective motion using internal agent beliefs gives insight into how social structure can influence, and be influenced by, the motion decisions of individuals. The model presented achieves cohesive collective motion in unbounded space with sensory radii spanning 12.5% of the initial group spread (*k_h_* = 0.125). The success of this model suggests that biasing agent motion using internal variables prevents diffusion in unbounded space, an important problem to solve for future real-world deployment of multi-agent and swarm robotic systems [7, 20]. The approach of modelling intelligent agents that use statistical inference methods to locally estimate and adaptively respond to mesoscopic system states opens up avenues for understanding agents who base their decisions on incomplete system information.

## Supplementary Material

Supplementary Materials

## Figures and Tables

**Table 1:** Variable definitions and parameter values used in simulations of the model.

Variable	Name	Definition or Value
*N*	Number of agents	100
*ρ*_0_	Initial density	0.05
*x_k_*	Position of agent *k*	−
*S_k_*	Social rank of agent *k*	−
*Ŝ_k_*(*x*)	Belief of agent *k*	−
Φ*_k_*	Model fidelity for belief of agent *k*	Eq.(1)
*V_k_* $\left(x_{k};x_{k}^{*},L\right)$	Potential acting on agent *k*	Eq.(2)
$x_{k}^{*}$	Potential center of agent *k*	Eq.(3)
*L*	Box potential radius	5, 10, 15,…, 120
*h*	Sensory radius	250, 500, 1000, 1500,…, 8000
*r*	Measurement probability	0.01
*P_jk_*(*S_j_, S_k_*)	Probability agents *j* and *k* interact	*H*_0_(Δ*S_max_−|S_j_− S_k_*|)
*Q_jk_*(*S_j_, S_k_*)	Probability agent *j* wins an interaction with *k*	*H*_1/2_(S*_j_*− S*_k_*)
Δ*S_max_*	Social interaction threshold	100
*δ* ^+^	Winning social update	1
*δ* ^−^	Losing social update	0
*t_c_*	Measurement waiting time	3 × 10^6^
