# Supplementary material for: Sociogenesis in Unbounded Space: Modelling Self-Organised Cohesive Collective Motion
Source: Phys Biol. Author manuscript; Available in PMC 2024 Aug 22. (PMC7616385; doi:10.1088/1478-3975/acc4ff)
Supplement: Supplementary Materials [file EMS198055-supplement-Supplementary_Materials.pdf]

Supplementary Materials: Sociogenesis in Unbounded Space: Modelling Self-Organised Cohesive Collective Motion

Zohar Neu*
Department of Engineering Mathematics, University of Bristol, Bristol, BS8 1TW, United Kingdom

Luca Giuggioli
Bristol Centre for Complexity Sciences, University of Bristol, Bristol, BS8 1UB, United Kingdom
(Dated: March 16, 2023)

I. PARAMETERS AND TIMING SCHEME FOR SIMULATIONS

Here we discuss details relating to simulations performed to obtain the results presented in the main text. All simulations were performed using a codebase written in the C++ programming language. An asynchronous time updating scheme is used in simulations to avoid parity issues arising from symmetries in agent behaviour. For example, when a synchronous updating scheme is used, two neighbouring agents may exchange positions without ever meeting on the same lattice site. This situation is avoided when using an asynchronous updating scheme. A flow diagram of the timing scheme is shown in Fig. 1. At each discrete time-step, t, the order in which the N agents move is randomised. When an agent, k, is selected to move, they firstly check if there are any other neighbouring agents on the same lattice site, x_k. If neighbours are detected, one neighbour, j, is selected at random as an interaction partner. An interaction then takes place between k and j with probability $P_{kj}(S_k, S_j)$. In simulation results presented in the main text, agents do not interact if they already had an interaction in the current time-step, or if the currently encountered neighbour was their most recent interaction partner. Note that these conditions mainly influence the system by slowing down the convergence rate of social ranks, and the same stability regimes form without them. The social ranks of the winner and loser are then modulated up and down by δ^+ and δ^- respectively. The initially selected agent, k, then performs a regression measurement with probability r. A high value for r enables agents to be more responsive to socio-spatial dynamics of their neighbours, but incurs a larger computational load for individual agents, and when performing simulations to study the system. On the other hand, when the measurement rate is too low, the rate of information updating is slow, resulting in agents being less responsive to system dynamics. Finally, the agent, k, updates its potential center, x_k^*, and takes a step according to its individual potential, $V_k(x; x_k^*, L)$. This completes the move of agent k, and the next agent in the randomised list then moves.

* Zohar.Neu@bristol.ac.uk

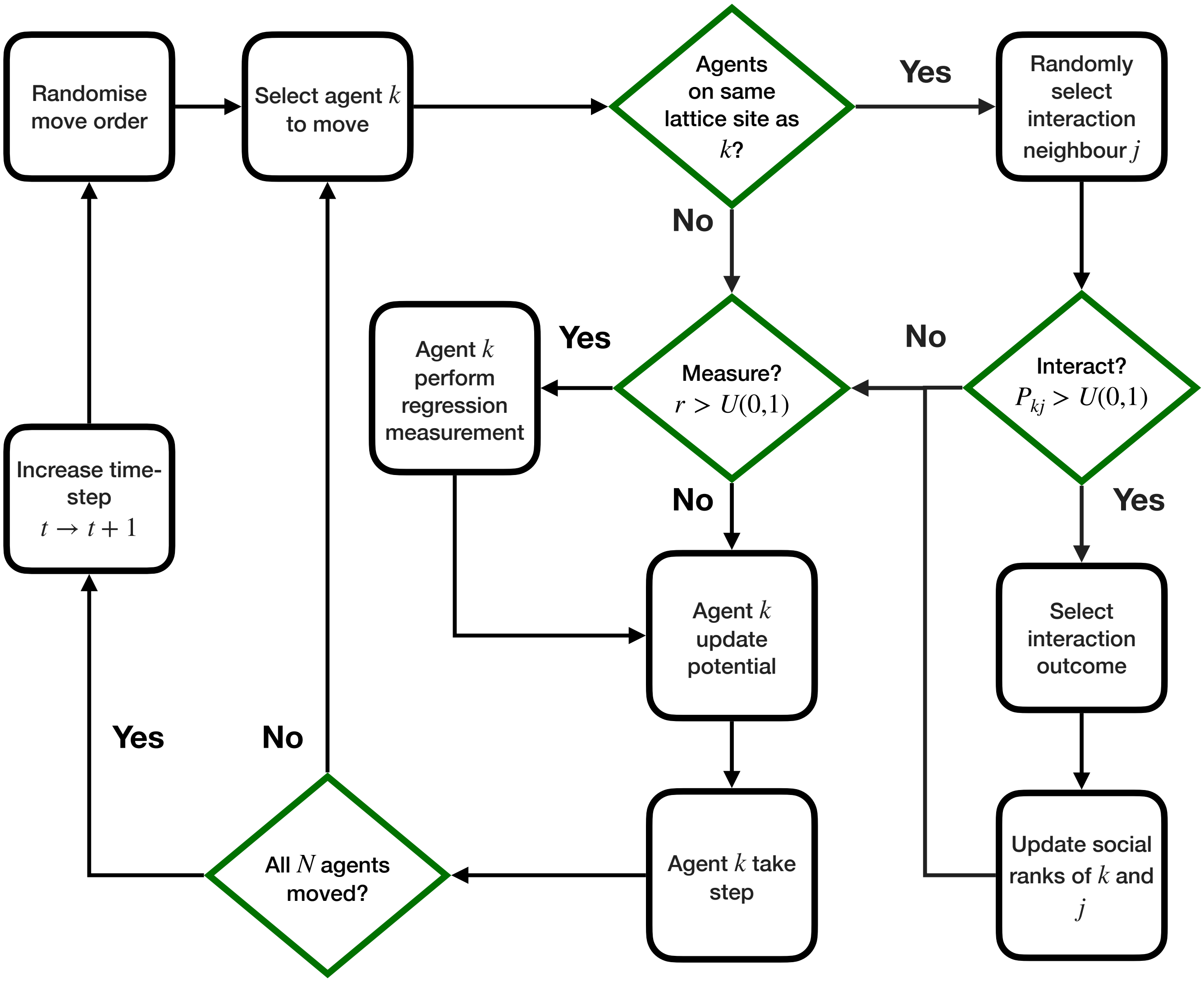

FIG. 1. Flow diagram for the timing scheme of simulations. The first time-step begins at the top-left corner of the flow diagram, and proceeds by following the direction of the arrows. The simulation ends when t reaches the pre-specified maximum number of time-step iterations.

II. BELIEF COMPUTATION PROCEDURE

A. Polynomial Regression

Agents compute their belief, $\widehat{S}_k(x)$, using polynomial regression, a linear regression method that assumes $\widehat{S}_k(x)$ to have the form of a polynomial of order u,

$$\widehat{S}_k(x) = \sum_{m=0}^{u-1} \beta_m x^m + \xi, \tag{S1}$$

where ξ is assumed to be a Gaussian white noise with variance σ^2. Such a belief may also be represented by the coefficient vector $\boldsymbol{\beta}_k = (\beta_0, \ldots, \beta_{u-1})^T$. When an agent, k, senses n neighbours, k obtains a data-set consisting of n observations $\{x_j, S_j\}_{j=1}^n$. Linear regression then consists of solving a set of n simultaneous equations, corresponding to Eq. (S1), for the coefficients in $\boldsymbol{\beta}_k$. Expressing the simultaneous equations in matrix form, this is equivalent to solving

$$\boldsymbol{S} = X\boldsymbol{\beta}_k + \boldsymbol{\xi}, \tag{S2}$$

where

$$\boldsymbol{S} = \begin{pmatrix} S_1 \\ S_2 \\ \vdots \\ S_n \end{pmatrix}, \quad X = \begin{pmatrix} 1 & x_1 & \dots & x_1^{u-1} \\ 1 & x_2 & \dots & x_2^{u-1} \\ \vdots & \vdots & \ddots & \vdots \\ 1 & x_n & \dots & x_n^{u-1} \end{pmatrix}, \quad \boldsymbol{\beta}_k = \begin{pmatrix} \beta_0 \\ \beta_1 \\ \vdots \\ \beta_{u-1} \end{pmatrix}, \quad \boldsymbol{\xi} = \begin{pmatrix} \xi_1 \\ \xi_2 \\ \vdots \\ \xi_n \end{pmatrix}, \tag{S3}$$

and for simplicity, it is assumed that each of the observations is subject to the same external process noise $\xi_j = \xi$, so that the problem is homoscedastic.

The ordinary least squares solution for the coefficient vector, $\boldsymbol{\beta}_k$, is found by minimising the residual sum of squares (RSS) between observations S_j and belief predictions $\widehat{S}_k(x_j)$

$$\text{RSS}(x_k) = \sum_j \left(S_j - \widehat{S}_k(x_j)\right)^2, \tag{S4}$$

whose minimum is found through differentiation with respect to the coefficient vector $\boldsymbol{\beta}_k$, and solving the resulting set of simultaneous equations. The ordinary least squares solutions is then given by [1]

$$\boldsymbol{\beta}_k = (\boldsymbol{X}^T\boldsymbol{X})^{-1}\boldsymbol{X}^T\boldsymbol{S}. \tag{S5}$$

B. Weighted Polynomial Regression

In the model presented in the main text, agents effectively perform local regression [1] to collectively estimate the social field, $S(x,t)$. Weighted polynomial regression is used in order for these estimates to be smooth, by scaling the contribution of each sensed neighbour, j, to the regression by a *distance dependent* weight factor w_j. The weight is defined according to the distance from each neighbour, j, located at x_j to the location of the measuring agent, x_k, through a smoothing kernel, $w_j = K_h(x_k, x_j)$, defined below. This smoothing kernel makes the agent sensing more local by reducing the contribution of data associated to agents who are further away from the agent performing the measurement. Furthermore, the smoothing kernel, K_h, can be considered as a model of agent perception, by defining the extent and strength of agent sensory capabilities.

The smoothing kernel, $K_h(x_k, x_j)$, has a general functional form [1]

$$K_h(x_k, x_j) = G\left(\frac{|x_k - x_j|}{h}\right), \tag{S6}$$

where different choices of G define different kernels, and h is the width of the smoothing neighbourhood, which in the present case is the sensory radius of the agent, k. Here, the tri-cube kernel [1]

$$G(t) = \begin{cases} (1-|t|^3)^3 & \text{if } |t| \le 1 \\ 0 & \text{otherwise}, \end{cases} \tag{S7}$$

is chosen as it offers compact support, which is desirable for computational convenience. If the support is not compact, agents are assumed to possess an infinite sensory range, which takes into account all other agents in the system for each computation.

Weighted regression computes $\widehat{S}_k(x)$ by minimising the residual sum of *weighted* squares between observations, S_j, and belief predictions, $\widehat{S}_k(x_j)$, given by

$$\text{RSWS}(x_k) = \sum_j W_{jj}\left(S_j - \widehat{S}_k(x_j)\right)^2, \tag{S8}$$

with normalised weights $W_{jj} = w_j/\sum_k w_k$. The weighted least squares solution is found by the same method as in the previous section [1]. In matrix form, this corresponds to computing the coefficients using the equation

$$\boldsymbol{\beta}_k = (\boldsymbol{X}^T\boldsymbol{W}\boldsymbol{X})^{-1}\boldsymbol{X}^T\boldsymbol{W}\boldsymbol{S}, \tag{S9}$$

where W is a diagonal matrix with elements $W_{jj} = w_j/\sum_k w_k$, so that $\sum_j W_{jj} = 1$.

C. Model Fidelity

The model fidelity, Φ_k, associated to a belief, $\widehat{S}_k(x)$, is used by agents as a local measure of fitting accuracy. The quality of the fit is evaluated by considering the amount of information in the sensed data that is *explained* by the computed belief, by comparing the *unweighted* residual sum of squares (RSS) to the *unweighted* total sum of weighted squares (TSS) that would result from a zero-order polynomial regression, $\widehat{S}_k(x) = \beta_0$, that is equivalent to an average of S_j,

$$\mathrm{TSS} = \sum_{j=1}^{n} \left(S_j - \bar{S}\right)^2, \tag{S10}$$

where $\bar{S}$ is the average of all S_j in the fitting domain. Supposing the agent, k, located at x_k senses n neighbours, the model fidelity is given by

$$\begin{aligned}\Phi_k &= 1 - \frac{RSS}{TSS}, \\ &= 1 - \frac{\sum_{j=1}^{n}\left(S_j - \widehat{S}_k(x_j)\right)^2}{\sum_{j=1}^{n}\left(S_j - \bar{S}\right)^2},\end{aligned} \tag{S11}$$

which results in a value $\Phi_k \in (-\infty, 1]$, with $\Phi_k \to 1$ indicating a good fit, and $\Phi_k \to -\infty$ indicating a worse fit. Mathematically, the model fidelity is equivalent to computing an unweighted coefficient of determination for a model fitted by weighted regression. Weighted regression model fitting is performed by giving a higher weight to data associated with neighbours that are closer to the sensing agent, and so the estimated model is "constrained" to fit these data points more closely. Hence, by construction, the weighted model may fit the data associated to far-away neighbours more poorly. The model fidelity, Φ_k, is evaluated by weighing all data equally, irrespective of distance from the sensing agent. Hence, unlike the coefficient of determination, it is possible that $\Phi_k < 0$ when the states of far-away agents diverge greatly from the estimated weighted regression model. Restricting agents from responding to estimates with $\Phi_k < 0$ by using a measurement waiting time t_c prevents agents from inferring incorrect motion decisions from their weighted regression model when it disagrees greatly with the states of far-away neighbours.

D. Problem Conditioning and Data Quality

For a regression measurement to be performed, the agent must have at least 3 neighbours within sensing distance, h. This is due to the fact that a second-order polynomial regression has 3 coefficients and so requires at least 3 data-points to fit. Given that a regression is performed, the weighted least squares regression solution in equation (S9) requires a square symmetric *moment matrix*, $X^T W X$, to be inverted. For this matrix to be invertible, the problem must be well conditioned, as poor conditioning of $X^T W X$ may lead to numerical instabilities when it is inverted [2]. The reciprocal condition number is a measure of how fluctuations in the input data change the resulting regression solution. This number depends on aspects of the moment matrix such as its rank and scale, with a low reciprocal condition number signifying poor conditioning. In simulations performed, a reciprocal conditioning number threshold, $\epsilon_c = 10^{-6}$, is defined, below which the moment matrix, $X^T W X$, is considered to be too poorly conditioned to invert.

E. Data Standardisation for Regression

Feature re-scaling [3] improves the regression problem conditioning by mapping the sensed values of x_j to a standardised scale. This allows for a single reciprocal conditioning number threshold, ϵ_c, to be used, since the scale of the input data has a direct effect on the reciprocal conditioning number. The regression method used assumes sensed data to have a normal distribution [1], and so standardisation is used in order to map the data, $x_j \to x'_j$, so that it has a standard normal distribution, $x'_j \sim \mathcal{N}(0, 1)$, according to

$$x'_j = \frac{x_j - \bar{x}}{\sigma_x}, \tag{S12}$$

where $\bar{x}$ and σ_x are the mean and standard deviation of the input data, x_j.

Due to the linearity of the regression, any re-scaling of the regression coefficients $\boldsymbol{\beta}_k \rightarrow \boldsymbol{\beta}'_k$ that results from re-scaling of data $x_j \rightarrow x'_j$ is reversible after computation of $\boldsymbol{\beta}'_k$. In the case when $\widehat{S}_k(x) = \beta_0 + \beta_1 x + \beta_2 x^2$, the coefficients for the original regression problem, $\boldsymbol{\beta}_k = (\beta_0, \beta_1, \beta_2)$, can be retrieved from the re-scaled coefficients, $\boldsymbol{\beta}'_k = (\beta'_0, \beta'_1, \beta'_2)$, through

$$\beta_0 = \beta'_0 - \frac{\bar{x}\beta'_1}{\sigma_x} + \frac{\bar{x}^2\beta'_2}{\sigma_x^2}, \tag{S13}$$

$$\beta_1 = \frac{\beta'_1}{\sigma_x} - \frac{2\bar{x}\beta'_2}{\sigma_x^2}, \tag{S14}$$

$$\beta_2 = \frac{\beta'_2}{\sigma_x^2}. \tag{S15}$$

III. NAVIGATION PROCEDURE

The potential center locations, $x_k^*(t)$, are found at each time-step, t, through the solution to the minimisation problem

$$x_k^*(t) = \underset{x}{\operatorname{argmin}} \left(|S_k(t) - \widehat{S}_k(x)| \right), \tag{S16}$$

which can be considered graphically as finding the points of intersection between the curve defined by a belief, $\widehat{S}_k(x)$, and the horizontal line at $\widehat{S}_k(x) = S_k$. When $\widehat{S}_k(x) = \beta_0 + \beta_1 x + \beta_2 x^2$, the solutions to Eq. (S16) are given by

$$x_\pm^* = \frac{-\beta_1 \pm \sqrt{\beta_1^2 - 4\beta_2(\beta_0 - S_k)}}{2\beta_2}, \tag{S17}$$

which yield zero, one or two real solutions. In case two solutions are found, the solution that is closest to the previous potential center location, $x_k^*(t-1)$, is chosen,

$$x_k^*(t) = \underset{x_+^*, x_-^*}{\operatorname{argmin}} \left\{ |x^*(t-1) - x_\pm^*| \right\}. \tag{S18}$$

If there are no real solutions, the location, x_k^*, that minimises Eq. (S16) is the maximum of the belief, $\widehat{S}_k(x)$, given by

$$x_k^*(t) = -\frac{\beta_1}{2\beta_2}. \tag{S19}$$

If $\beta_2 = 0$, meaning that the agent had performed a first-order polynomial regression, the solution to Eq. (S16) is the location, $x_k^*(t)$, which solves

$$S_k(t) = \beta_0 + \beta_1 x_k^*(t), \tag{S20}$$

which is given by

$$x_k^*(t) = \frac{S_k(t) - \beta_0}{\beta_1}. \tag{S21}$$

Finally, a threshold, $\epsilon_\beta = 10^{-4}$, is used, such that the potential center, $x_k^*(t)$, is only updated when $|\beta_1| > \epsilon_\beta$. This is done in order to mitigate large fluctuations in computed potential center locations resulting from near-constant beliefs, $\widehat{S}_k(x)$, i.e. when $\beta_2 = 0$ and $\beta_1 \approx 0$. This may occur when all agents have similar social ranks, S_k, for example in early stages of the formation, or near minima of $S(x,t)$ when agents fit a first-order polynomial regression. In both cases, the low magnitude gradient leads to potential centers, x_k^*, that can be far away from the current location of the agent, removing them from the group. This parameter requires tuning, since too high a value leads to unresponsive agents, whereas too low a value leads to agents that are too responsive to low magnitude gradients.

IV. INTEGRATED SIGMOID SHAPE FUNCTION

The integrated sigmoid function

$$F(x;\alpha,m,K) = -\frac{2m}{\alpha}\ln\left(\cosh\left(\frac{\alpha x}{2}\right)\right) + K,$$
$$= -\frac{2m}{\alpha}\ln\left(1+e^{-\alpha x}\right) - mx + K, \tag{S22}$$

is obtained by integrating

$$f(x) = -m\tanh\left(\frac{\alpha x}{2}\right),$$
$$= -m\frac{e^{-\alpha x}-1}{e^{-\alpha x}+1}, \tag{S23}$$

with respect to x, where K is obtained as a constant of integration. It can be shown that the integrated sigmoid in Eq. (S22) interpolates between an absolute value function ($\alpha \approx 0$) and a quadratic function ($\alpha \to \infty$). Using L'Hospital's Rule as $\alpha \to \infty$,

$$\lim_{\alpha\to\infty} F(x;\alpha,m,K) = \lim_{\alpha\to\infty} -2m\frac{\ln(1+e^{-\alpha x})}{\alpha} - mx + K, \tag{S24}$$
$$= \lim_{\alpha\to\infty} -2m\frac{-xe^{-\alpha x}}{1+e^{-\alpha x}} - mx + K. \tag{S25}$$

When $x > 0$, the first term tends to zero. When $x < 0$, the first term tends to $2mx$. Hence,

$$\lim_{\alpha\to\infty} F(x;\alpha,m,K) = -m|x| + K. \tag{S26}$$

When $\alpha \approx 0$, $F(x;\alpha,m,K)$ may be expanded around $\alpha = 0$ using the Taylor series

$$\ln(1+e^{y}) = \ln(2) + \frac{y}{2} + \frac{y^2}{8} + O(y^4), \tag{S27}$$

where $y = -\alpha x$. Simplifying the resulting expression, we obtain the quadratic function

$$F(x;\alpha,m,K) \approx -\frac{\alpha m x^2}{4} + K - \frac{2m}{\alpha}\ln(2) \tag{S28}$$

for $\alpha \approx 0$.

However, α alone is not enough to classify the different regimes due to the additional dependence of $F(x;\alpha,m,K)$ on m and K, as shown in Fig. 2. Namely, values may be chosen for m and K which counteract the influence that α has in its asymptotic regimes, for example sending $m \to \infty$ or $K \to \infty$ in Eq. (S22). For this reason, the variable $J = \alpha K/m - \ln(4)$ is introduced, which captures this multiple asymptotic dependence. This form of J is chosen because it also determines the number of real roots of Eq. (S22), and so determines the region of validity for the fit.

The roots, $x^*_\pm$, of the integrated sigmoid are found by solving $F(x;\alpha,m,K) = 0$. Letting $z = \alpha x$ in Eq. (S22), this reduces to

$$0 = -2\ln(1+e^{-z}) - z + \frac{K\alpha}{m}. \tag{S29}$$

Re-arranging for z, exponentiating, and taking a square root both sides results in

$$z + \ln((1+e^{-z})^2) = \frac{K\alpha}{m}, \tag{S30}$$
$$\iff e^{z}(1+e^{-z})^2 = e^{\frac{K\alpha}{m}}, \tag{S31}$$
$$\iff e^{z/2}(1+e^{-z}) = e^{\frac{K\alpha}{2m}}, \tag{S32}$$
$$\iff e^{z/2} + e^{-z/2} = e^{\frac{K\alpha}{2m}}. \tag{S33}$$

Letting $s = e^{z/2}$,

$$s + s^{-1} = e^{\frac{K\alpha}{2m}}, \tag{S34}$$

$$\iff s^2 - e^{\frac{K\alpha}{2m}} s + 1 = 0, \tag{S35}$$

from which it follows that

$$s = \frac{e^{\frac{K\alpha}{2m}} \pm \sqrt{e^{\frac{K\alpha}{m}} - 4}}{2}. \tag{S36}$$

With $z = \ln(s^2) = 2\ln(s)$, it follows that

$$z = 2\ln\left(\frac{e^{\frac{K\alpha}{2m}} \pm \sqrt{e^{\frac{K\alpha}{m}} - 4}}{2}\right), \tag{S37}$$

$$= \pm 2\ln\left(\frac{e^{\frac{K\alpha}{2m}} + \sqrt{e^{\frac{K\alpha}{m}} - 4}}{2}\right). \tag{S38}$$

Recalling that $z = \alpha x$, the two roots of the integrated sigmoid are given by

$$x^*_\pm = \frac{\pm 2}{\alpha}\ln\left(\frac{e^{\frac{K\alpha}{2m}} + \sqrt{e^{\frac{K\alpha}{m}} - 4}}{2}\right). \tag{S39}$$

This solution naturally leads to a definition of J. Eq. (S39) has real solutions if

$$e^{\frac{K\alpha}{2m}} + \sqrt{e^{\frac{K\alpha}{m}} - 4} > 0, \tag{S40}$$

and if

$$e^{\frac{K\alpha}{m}} - 4 \geq 0, \tag{S41}$$

or equivalently

$$\frac{K\alpha}{m} - \ln(4) \geq 0, \tag{S42}$$

The inequality in Eq. (S40) is true if the inequality in Eq. (S42) is true. Hence, letting $J = K\alpha/m - \ln(4)$, this defines a lower bound such that the integrated sigmoid has real roots only when $J \geq 0$. The value $J > 0$ corresponds to two real solutions, whereas $J = 0$ corresponds to one real root where the function maximum touches the $x-$axis, and the rest of the function is negative. In this latter case, the integrated sigmoid no longer defines a valid fit for the configuration of agents.

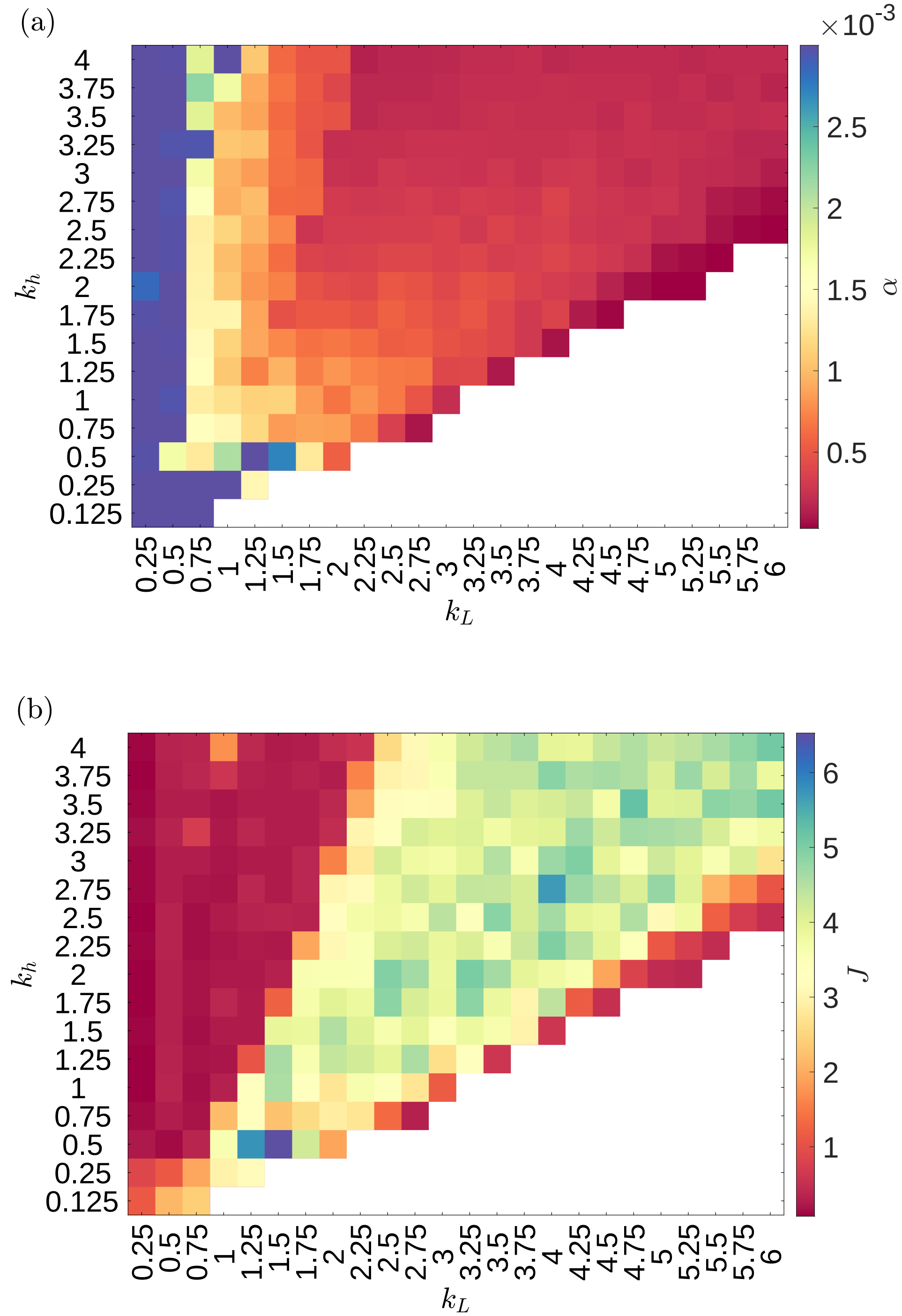

FIG. 2. **(a)** The shape regimes of the integrated sigmoid in Eq. (S22) cannot be classified using α alone. **(b)** Using $J = \alpha K/m - \ln(4)$ enables us to distinguish the different macroscopic configurations between the different regimes. In both cases, the unstable regime has not been considered, because here agents in the system expand away from each other to such an extent that the integrated sigmoid no longer provides an informative fit. Data corresponds to the results presented in Fig. 3 of the main text.

V. MEASUREMENT WAITING TIME EXPERIMENTS FROM VARYING INITIAL CONDITIONS

To test the efficacy of the two-stage dynamics, we investigate the ability of the system to converge to a concave annular state from a variety of initial conditions when $t_c = 0$ and when $t_c = 3 \times 10^6$. The system is considered to have converged at time t^* when the socio-spatial correlation first passes below a pre-defined threshold value, $C(t^*) < C^* = -0.97$. The 'W'-initial condition, shown in the inset of Fig. 3(a), is defined by an initial social rank profile

$$S_0^W(z_1, m) = \Delta S_{max} |z_1 - |N/2 - m| |, \tag{S43}$$

where $m = 1, \ldots, N$ is the ranked initial position of each agent from left to right and $z_1 = 0, \ldots, N/2$ interpolates between initial convex ($z_1 = 0$) and concave ($z_1 = N/2$) annular configurations.

We find that for both conditions of t_c, as z_1 is decreased from $z_1 = N/2$, there exists a threshold value, z_1^*, below which the system no longer converges to a concave annular state. This value of z_1^* is lower for $t_c = 3 \times 10^6$ than for $t_c = 0$, indicating that the two-stage dynamics increases the likelihood of reaching the desired goal state. This is illustrated in the insets of Fig. 3(a), which show the system time evolution with and without t_c for a selected value of $z_1 = 30$. When $t_c = 0$, the system remains frozen in the initially defined state and the presence of the radially increasing (convex) edges, facing away from the group center, is maintained. Motion biased by beliefs of agents located at the minima of $S(x, t)$ quickly pushes the system apart so that agents cannot interact with or sense their neighbours. The system becomes locked into the initial configuration, which remains for the duration of simulations. The presence of a measurement waiting time, $t_c > 0$, enables agents to have more interactions in the minima of the configuration. This differentiation in social ranks leads to a destabilisation of the radially increasing edges, which re-configure themselves into radially decreasing, concave edges.

The 'W'-initial condition experiments suggest that the existence of concave, radially decreasing edges at early times is a precursor for the system to reach the desired cohesive pattern at long times. To confirm this hypothesis, we define the concave edge initial condition by initialising the social ranks with $S_0^C(z_2, m) = \Delta S_{max}(N/2 - |N/2 - m|)$ when the ranked position $m = 1, \ldots, N$ is at least z_2 agents away from the center ($m < z_2$ or $m > N - z_2$), and otherwise the agent social rank is randomly sampled from a uniform integer distribution $S_0^C(z_2, k) \sim U(0, N\Delta S_{max}/2)$, where $S_k = N\Delta S_{max}/2$ is the theoretical maximum social rank in the concave annular state. As shown in Fig. 3(b), here a threshold value, $z_2 = z_2^*$, also exists, below which the system does not converge to an annular state. The value of z_2^* is decreased when $t_c > 0$, illustrating that the two-stage dynamics allows the system to reach the goal state from a larger perturbation.

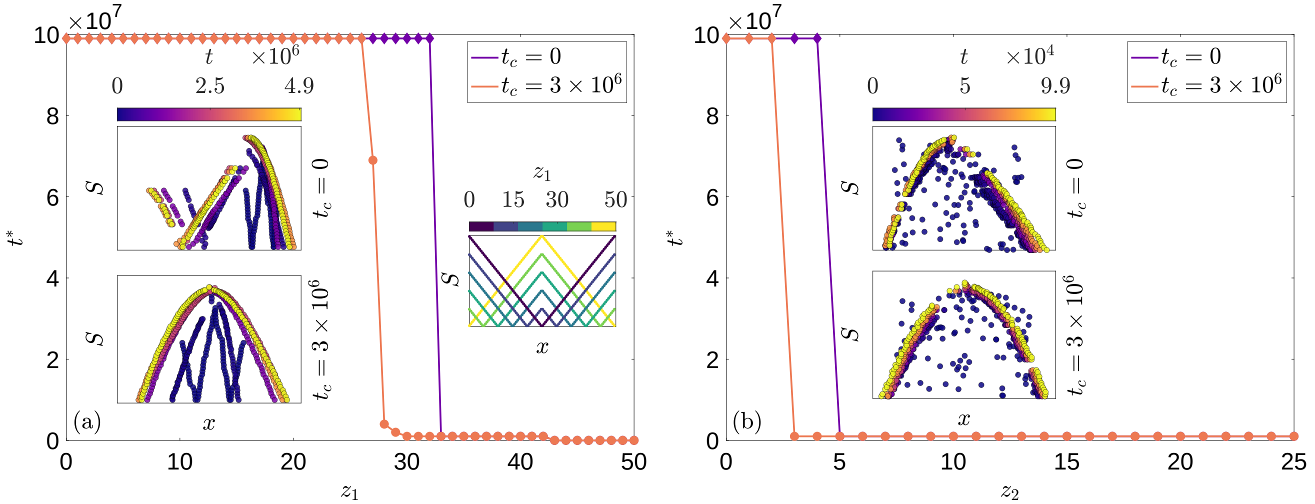

FIG. 3. (Colour online) Convergence times, t^*, from different initial conditions with and without a measurement waiting time, t_c. **(a)** 'W'-initial condition, $S_0^W(z_1, m)$. Insets correspond to $z_1 = 30$. **(b)** Concave edge initial condition, $S_0^C(z_2, m)$. Insets correspond to $z_2 = 3$. In this case, the configurations obtained for $t_c = 0$ are concave but not radially symmetric. In both cases, the inclusion of $t_c > 0$ increases the range of initial condition states that converge to a concave annular state, defined by a socio-spatial correlation $C(t) < -0.97$. Simulation parameters are the same as those in Fig. 2 of the main text, with $k_h = 0.5$ and $k_L = 1$, and results are averaged over 50 Monte Carlo realisations.

VI. SUPPLEMENTAL RESULTS

A. Social Convergence

The systems exhibits convergence to a concave annular state in the stable Full Sensing and Partial Sensing regimes, as shown in Fig. 4(a). It can be seen that parameter values with lower magnitude socio-spatial correlation are found in the Unstable regime, where the sensed proportion of agents is below $n_s(t) < 0.06$, which in simulations corresponds to approximately 6 sensed agents. Below this value, the collective agent motion resulting from computed beliefs, $\widehat{S}_k(x)$, becomes unstable. If such instabilities lead small groups of agents to move away from the group, the group center of mass is shifted, thereby reducing the magnitude of the socio-spatial correlation.

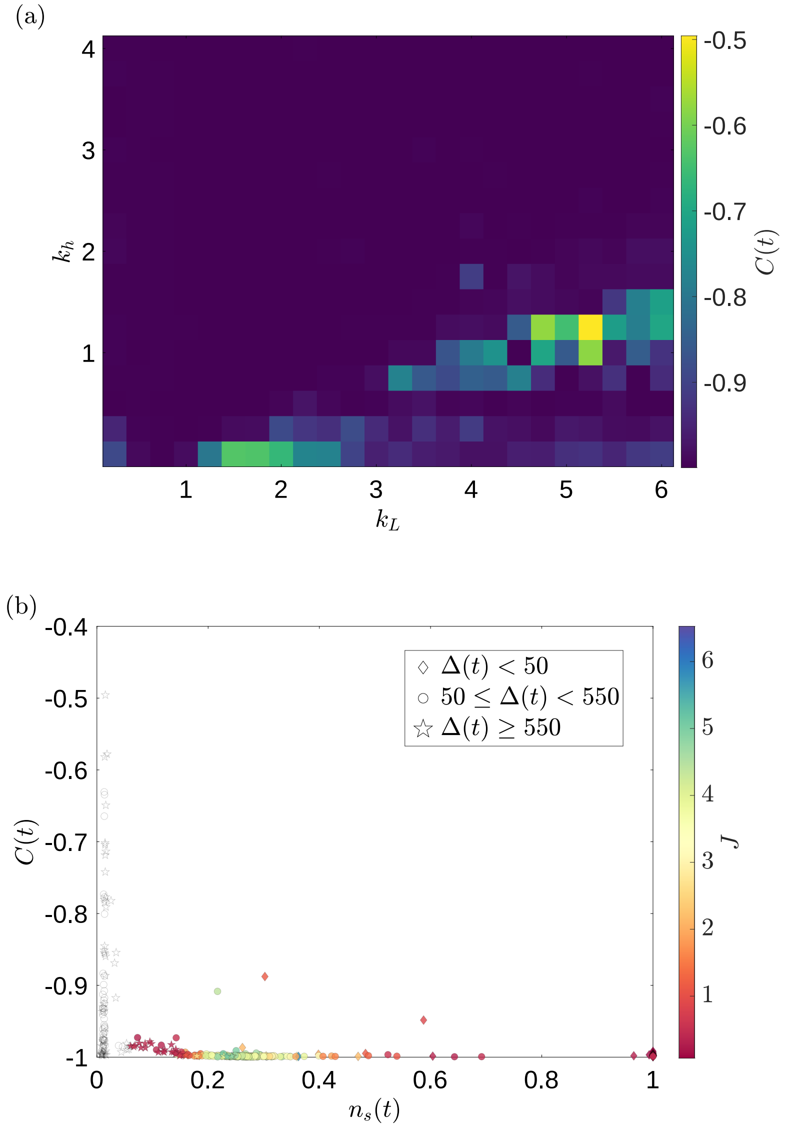

FIG. 4. **(a)** Socio-spatial correlation, $C(t)$, in the (k_L, k_h)-state-space plotted after $t = 10^9$ steps. Lower magnitude values of $C(t)$ are obtained in the Unstable regime, where unstable expansion leads some agents to move away from the group. This shifts the group center of mass, thereby decreasing the socio-spatial correlation. **(b)** Scatterplot of SSC values plotted against the average proportion of sensed neighbours $n_s(t)$. Points are coloured by the corresponding value of J and their shape is determined by the mean nearest-neighbour distance $\Delta(t)$. Colourless points are those with $n_s(t) < 0.06$ and correspond to the Unstable regime. Data corresponds to the results presented in Fig. 3 of the main text.

B. Long time stationarity of system

1. *Mean-Squared-Displacement Power-Law Fitting*

The spatial dispersion of the system can be quantified using the mean-squared displacement (MSD). When agent dynamics are not independent, they may exhibit inhomogeneous diffusivity and transport behaviour. In this case, it is more informative to use the mean-squared-distance to the group center of mass,

$$\nu(t) = \left\langle \sum_{k=1}^{N} |x_k(t) - \bar{x}(t)|^2 \right\rangle, \tag{S44}$$

where $\bar{x}(t)$ represents the group center of mass and $x_k(t)$ is the position of the k-th agent at time t in each realisation. This measure is only affected by the relative distance of agents away from the center of mass, and can be used to account for the relocation of agents early on in the process.

In the long time limit, $t \to \infty$, the MSD in Eq. (S44) assumes a power-law relationship

$$\nu(t) = Bt^{\gamma_\nu}, \tag{S45}$$

where the power-law coefficient, γ_ν, distinguishes between different dynamical regimes. A coefficient of $\gamma_\nu = 1$ signifies diffusive motion, while $\gamma_\nu < 1$ and $\gamma_\nu > 1$ signify sub-diffusive and super-diffusive motion, respectively. The MSD is also used for identifying confinement behaviour [4]. When an agent becomes confined to a certain region of space, $x(t) \in [a, b]$, the MSD asymptotically saturates to a constant value. To find this relationship from data, the natural logarithm is taken of the MSD,

$$\ln(\nu(t)) = \ln(B) + \gamma_\nu \ln(t). \tag{S46}$$

and a linear fit is performed for large t to find γ_ν. This is plotted in Fig. 5, where it can be seen that the stable regimes result in power-law coefficients with magnitude $|\gamma_\nu| < 10^{-1}$, while the unstable regimes are either still expanding with $|\gamma_\nu| > 1$ or have already expanded so that agents no longer have enough neighbours to make measurements, leading them to stop updating their potential centers.

2. *Mean Nearest-Neighbour Distance Power-Law Fitting*

Similarly to the mean-squared displacement (MSD) measure, the mean nearest-neighbour distance assumes a power law relationship

$$\Delta(t) = At^{\gamma_\Delta}, \tag{S47}$$

in the long time limit, $t \to \infty$ [5]. The power-law coefficient, γ_Δ, distinguishes between different behavioural regimes of the system, with lower magnitude values of $\gamma_\Delta < 1$ corresponding to slower spatial expansion in the system, and $\gamma_\Delta = 0$ when the system attains a confined state. This is plotted in Fig. 6, along with the corresponding curves for the mean nearest-neighbour distance. As in Fig. 5, the fitted power-law coefficient distinctly shows the stable and unstable regions.

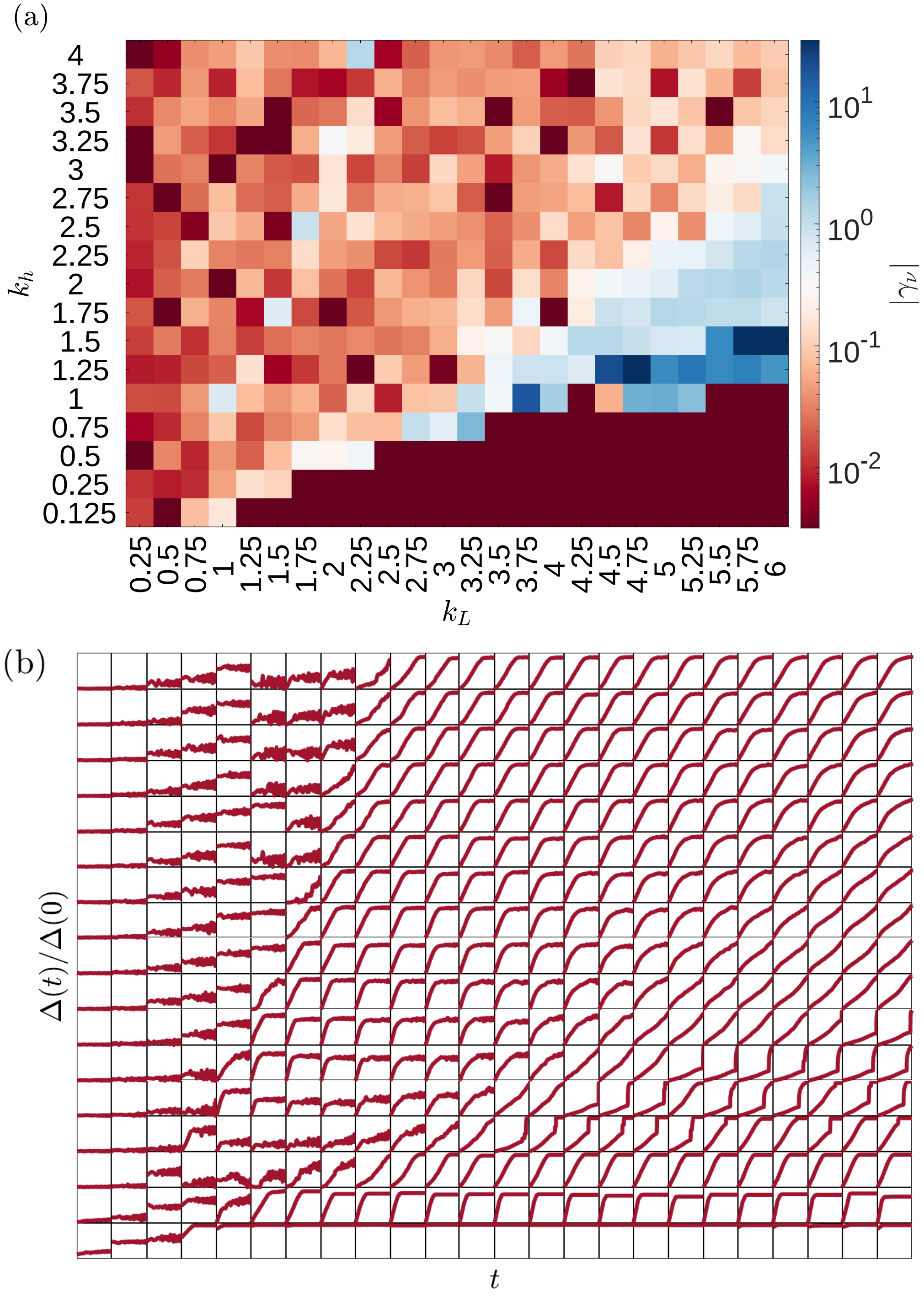

FIG. 5. **(a)** Fitted power-law coefficient, $|\gamma_\nu|$, for the mean-squared-displacement, $\nu(t)$, at time $t = 10^9$. The absolute value is taken so that data can be plotted on a logarithmic scale when γ_ν is close to zero and negative. Higher power-law coefficient values are found on the cusp between the Full and Partial Sensing regimes, where the system exhibits two possible expansion states and has not yet reached equilibrium. The power-law coefficient was fitted between $t = 6.45 \times 10^8$ and $t = 9.95 \times 10^8$. **(b)** Corresponding MSD curves plotted on a log-log scale for each parameter-pair k_L and k_h. On each vertical axis, the MSD, $\nu(t)$, normalised by the initial value is plotted, while time, t, is plotted on the horizontal axes. Data corresponds to the results presented in Fig. 3 of the main text.

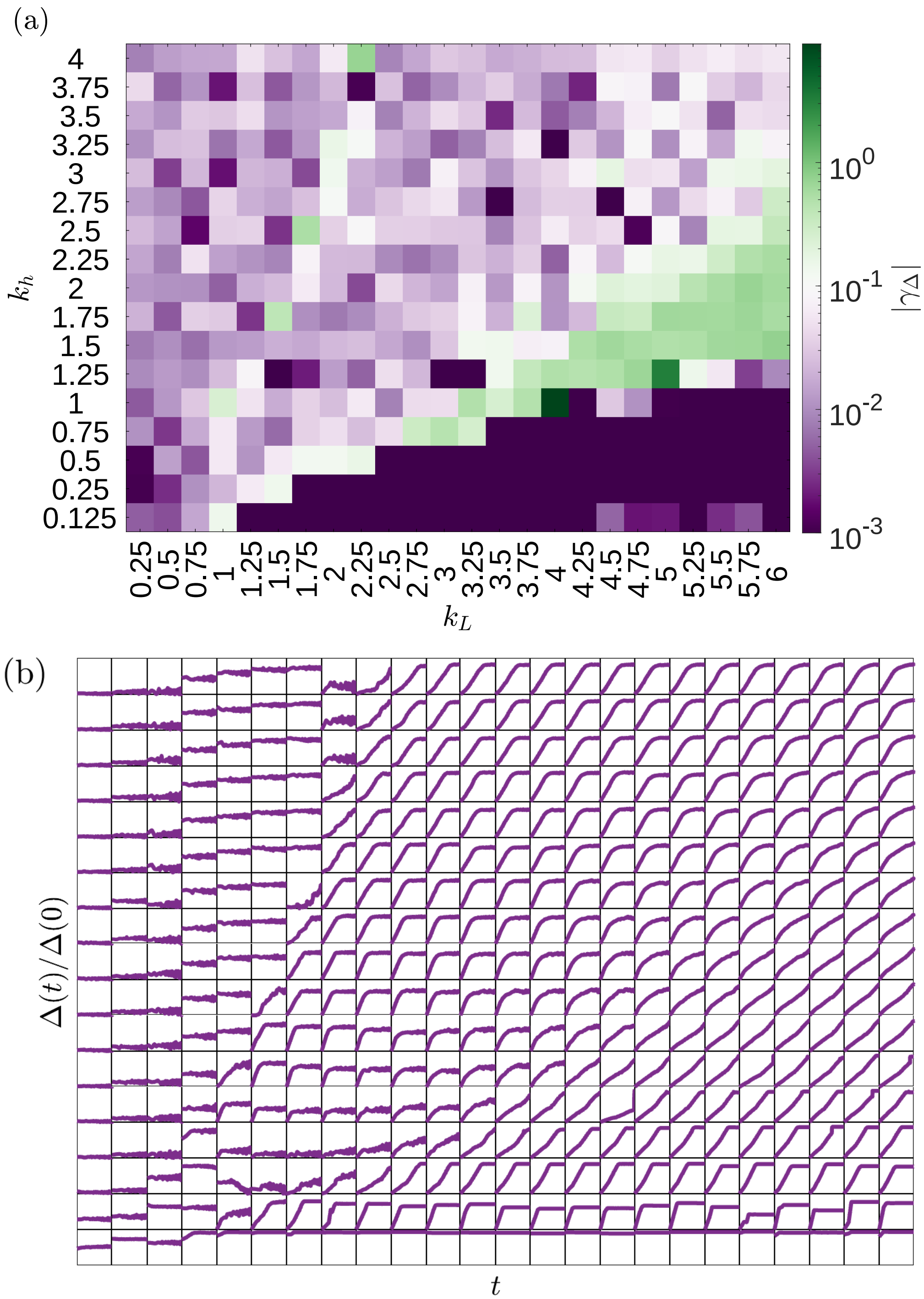

FIG. 6. **(a)** Fitted power-law coefficient, $|\gamma_\Delta|$, of the mean nearest-neighbour distance, $\Delta(t)$, at time $t = 10^9$. The absolute value is taken so that data can be plotted on a logarithmic scale. As in Fig. 5, higher magnitude values of $|\gamma_\Delta|$ are found on the cusp between the Full and Partial Sensing regimes. Power-law coefficient fitting times are the same as in Fig. 5. The colourbar has a lower limit of $|\gamma_\Delta| > 10^{-3}$ to aid visualisation, as values in the Unstable regime reach $|\gamma_\Delta| = 10^{-15}$. **(b)** Normalised mean nearest-neighbour distance for each parameter-pair, k_L and k_h. Vertical axes show $\Delta(t)$ normalised by the initial value, while time, t, is plotted on the horizontal axes (log-log scale). Data corresponds to the results presented in Fig. 3 of the main text.

[1] T. Hastie, R. Tibshirani, and J. Friedman, *The elements of statistical learning: data mining, inference, and prediction* (Springer Science & Business Media, 2009).
[2] D. A. Belsley, E. Kuh, and R. E. Welsch, *Regression diagnostics: Identifying influential data and sources of collinearity*, Vol. 571 (John Wiley & Sons, 2005).
[3] J. Han, J. Pei, and M. Kamber, *Data mining: concepts and techniques* (Elsevier, 2011).
[4] A. H. Robles and L. Giuggioli, Phase transitions in stigmergic territorial systems, Physical Review E **98**, 042115 (2018).
[5] M. Zumaya, H. Larralde, and M. Aldana, Delay in the dispersal of flocks moving in unbounded space using long-range interactions, Scientific reports **8**, 1 (2018).
